# Eugenol derivatives: synthesis, characterization, and evaluation of antibacterial and antioxidant activities

**DOI:** 10.1186/s13065-018-0407-4

**Published:** 2018-04-03

**Authors:** Francisco Felipe Maia da Silva, Francisco José Queiroz Monte, Telma Leda Gomes de Lemos, Patrícia Georgina Garcia do Nascimento, Alana Kelly de Medeiros Costa, Luanda Misley Mota de Paiva

**Affiliations:** 1 Instituto Federal de Educação, Ciência e Tecnologia do Rio Grande do Norte (IFRN), RN 233, Km 02 N°999, Chapada do Apodi, Apodi, RN 59700-000 Brazil; 2 Programa de Pós-Graduação em Química da Universidade Federal do Ceará (UFC), Avenida Humberto Monte, S/N, Campus do pici, Fortaleza, CE 60455-900 Brazil

## Abstract

Eugenol is the major component of clove essential oil and has demonstrated relevant biological potential with well-known antimicrobial and antioxidant action. Therefore, this work carried out the synthesis, purification, characterization, and evaluation of the antioxidant and antibacterial potential of 19 eugenol derivatives. The derivatives were produced by esterification reactions in the hydroxyl group (−OH) of eugenol with different carboxylic acids and also by addition reactions in the double bond of the allyl group. The derivatives had a promising antibacterial potential, including a lower minimum inhibitory concentration of 500 μg/mL than eugenol (1000 μg/mL). In addition, the derivatives were active against bacterial strains (*Escherichia coli*, *Staphylococcus aureus*) that eugenol itself showed no activity, thus increasing the spectrum of antibacterial action. As for the antioxidant activity, it was observed that the derivatives that involved esterification reactions in the hydroxyl group (−OH) of the eugenol molecule’s phenol resulted in a significant reduction of the antioxidant action (IC_50_ > 100 μg/mL) when compared with the eugenol precursor molecule (IC_50_ = 4.38 μg/mL). On the other hand, the structural changes located in the double bond affected much more smoothly the capacity of capturing radicals than the starting molecule, also being obtained derivatives with proximal antioxidant capacity (IC_50_ = 19.30 μg/mL) to commercial standards such as Trolox (IC_50_ = 16.00 μg/mL).

**Electronic supplementary material:**

The online version of this article (10.1186/s13065-018-0407-4) contains supplementary material, which is available to authorized users.

## Introduction

Molecular modification in structures of biologically active substances that occur naturally is one of the main strategies to enhance healthy biological effects, as well as to reduce eventual side effects [1–3]. In 1998, it was estimated that 60% of the antitumor and anti-infective drugs that entered the market or under clinical trial originated from natural products [4] via structural modifications. More recent data (December 2014) show that of the 237 drugs used as anti-infectious agents (antibacterial, antifungal, parasitic, and antiviral), excluding vaccines, recognized by public health agencies in the world, 138 (approximately 58.30%) are products of natural origin or derived from natural products. Thus, it is clear that this is a line of research with great potential for obtaining new drugs [5].

Eugenol, a natural substance used as a target molecule for the manufacture of bioactive compounds, was first isolated in 1929 and its commercial production began in 1940 in the United States. It can be produced synthetically; however, it is mainly extracted from *Ocimum tenuiflorum*, *Cassia fistula*, *Zieria smithii*, and *Pimenta racemosa*. Classified as a phenylpropanoid of the allyl-phenol type, eugenol is a pale yellow oil with clove odor and spicy taste. With numerous applications in the pharmaceutical, food, agricultural, and cosmetics industries [6, 7], it showed promising antimicrobial and antioxidant effects [8–12], when evaluated against the fungi *Cladosporium* spp. [13]. Other activities are reported in literature, such as antiviral [14, 15], anti-inflammatory [16], and inhibitor of platelet aggregation [17]. In addition, its anti-Leishmania activity together with its low cytotoxicity qualifies it as a promising source of new leishmanicidal [18].

This broad spectrum of biological activity makes eugenol a target molecule for structural modifications in order to produce substances with therapeutic properties. Currently, among the various clinical pathologies, bacterial infections and cellular oxidation stand out, both with serious implications for public health. Due to the acquisition of resistance and the mutational capacity of microorganisms, commercial antibiotics are in many cases incapable of fighting bacterial proliferation, resulting in failures in the treatment associated with multiresistant bacteria. Consequently, bacterial resistance has become a global concern regarding public health [19–21].

Aerobic organisms have the ability to produce free radicals that, in excess, can initiate chain reactions that damage cells or cause death to the latter. As a consequence, several diseases arise, especially cardiovascular and neurodegenerative diseases. The fight against the effects resulting from the production of free radicals has been the dietary use of antioxidant substances with a significant effect in the prevention of these diseases [22–28]. Eugenol, according to reports in literature, combats oxidative stress with beneficial effects on health.

Thus, in view of the broad spectrum of biological activities of eugenol, the present study aimed to obtain its derivatives by means of esterification and addition reactions. All were submitted to the evaluation of the antibacterial and antioxidant potential with very interesting results.

## Results and discussion

**Scheme 1:**
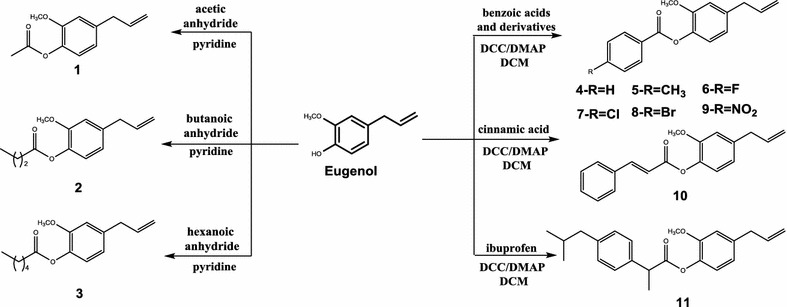
Eugenol derivatives (**1–11**) through reactions in the hydroxyl groups

**Scheme 2:**
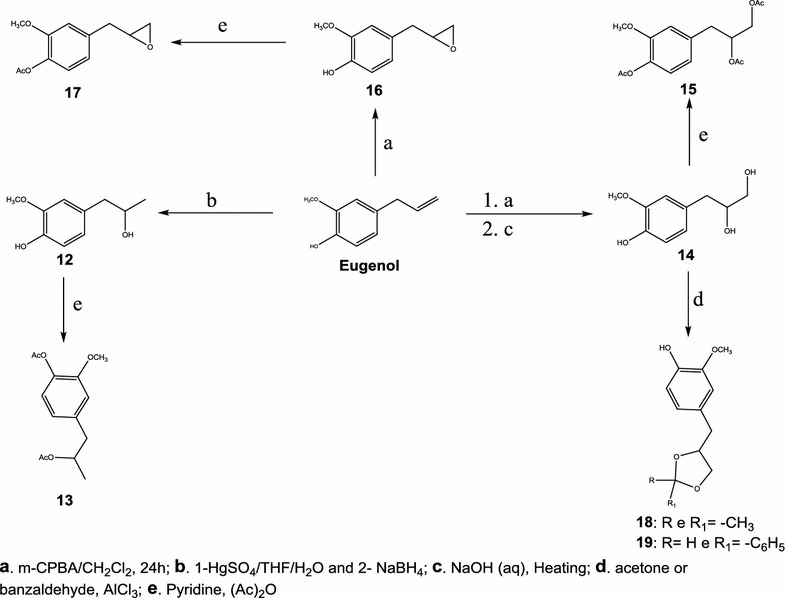
Eugenol derivatives (**12–19)** through reactions in the double bond

Structural modifications from eugenol, carried out on the hydroxyl group and the olefinic bond, were represented in reaction Schemes 1 and 2, respectively.

Among the compounds prepared, 3, 13, 15, 17, 18, 19 are unpublished in literature. Compounds 1, 2, 4, 5, 6, 7, 8, 9, 10 and 11 have already been prepared in previous works [11, 29–31], however through different synthetic routes.

Evaluation of the antibacterial activity of eugenol derivatives using the inhibition zone technique, measured in millimeter (mm), demonstrates the potential for inhibition of microbial growth by a given substance. According to literature, substances with inhibition halos less than 7 mm, greater than 7 and less than 16 mm, and greater than 16 mm are considered inactive, moderately active, and antibacterial potential, respectively [32, 33]. The results presented in Table 1 show the inhibition zones (halos) presented by the eugenol derivatives against six bacterial strains.

**Table 1:** Effect of eugenol derivatives against six bacterial strains

Compound	Zone of inhibition (mm)					
	*Pseudomonas aeruginosa*	*Escherichia coli*	*Staphylococcus aureus*	*Streptococcus*	*Klebsiella pneumoniae*	*Bacillus cereus*
**1**	0	0	0	0	0	0
**2**	0	0	0	0	0	0
**3**	0	0	0	0	0	0
**4**	0	0	0	0	0	12
**5**	0	0	0	6	0	12
**6**	0	0	0	0	0	0
**7**	0	0	0	0	0	0
**8**	0	12	6	0	0	0
**9**	0	0	0	11	0	0
**10**	0	0	0	12	6	0
**11**	0	0	0	0	0	0
**12**	0	0	0	10	0	0
**13**	0	0	0	12	0	8
**14**	0	0	0	9	0	0
**15**	0	12	0	10	6	0
**16**	0	0	10	10	15	20
**17**	6	0	0	10	6	12
**18**	0	0	0	9	0	0
**19**	0	0	0	0	0	0
Methyl eugenol	0	0	0	0	0	0
Eugenol	12	0	0	6	11	12
Isoeugenol	0	0	12	0	0	0
Tetracycline	0	10	20	10	9	10

According to the results (Table 1), acetylation of eugenol did not result in any benefit, since esters **1**, **2**, and **3** showed no antibacterial action against any of the strains. Also, esterification with benzoic acid and its *p*-substituted derivatives yielded derivatives **4**–**9** which exhibited random but still insufficient activities relative to eugenol itself.

Regarding derivatives **10**–**18** resulting from double bond addition, **10**–**14**, **17**, and **18** showed random and insufficient activities relative to eugenol. However, according to the classification shown above, derivative **16** showed a strong antibiotic effect against *Bacillus cereus* and a moderate effect against *Staphylococcus aureus*, *Streptococcus*, and *Klebsiella pneumoniae*, but was inactive in cases of *Pseudomonas aeruginosa* and *Escherichia coli*. It was of interest to observe that compound **16**, except for the bacterium *P. aeruginosa*, showed greater activity than eugenol itself. In the case of derivative **15**, a triacetyl derivative, it is of particular interest to highlight the high antibacterial activity (inhibition halo 12) against *E. coli* relative to eugenol (inhibition halo 0). By contrast, 1**5** was inactive (inhibition halo 0) against *P. aeruginosa*, whereas eugenol was active (inhibition halo 12).

A recent work [34] revealed antimicrobial activity for eugenol against strains *E. coli* and *S. aureus*, exhibiting inhibition halos with diameters of 9.25 and 7.75 mm, respectively. Although the results in the present study did not show activity for these strains, it is worth mentioning that the amounts (3 mg) applied in the first one were 13 times higher than those (0.2 mg) used in the present study. Of the derivatives priorly mentioned, those with inhibition halos greater than 6 mm were subjected to microdilution tests to determine the minimum inhibitory concentration (MIC) which prevents visible growth of the bacteria. Table 2 shows the results for derivatives **4**–**18** expressed in μg/mL.

**Table 2:** Minimum inhibitory concentration (MIC) presented by derivatives 1–19 against different bacterial strains

Compound	Minimum inhibitory concentration MIC (µg/mL)					
	*Pseudomonas aeruginosa*	*Escherichia coli*	*Staphylococcus aureus*	*Streptococcus*	*Klebsiella pneumoniae*	*Bacillus cereus*
**4**	NA	NA	NA	NA	NA	NA
**5**	NA	NA	NA	1000	NA	NA
**8**	NA	500	1000	NA	NA	NA
**9**	NA	NA	NA	1000	NA	NA
**10**	NA	NA	NA	1000	NA	NA
**12**	NA	NA	NA	1000	NA	NA
**13**	NA	NA	NA	1000	NA	NA
**14**	NA	NA	NA	NA	NA	NA
**15**	NA	1000	NA	NA	NA	NA
**16**	NA	NA	NA	NA	500	500
**17**	NA	NA	NA	NA	NA	1000
**18**	NA	NA	NA	NA	NA	NA
**19**	NA	NA	NA	NA	NA	NA
Eugenol	1000	NA	NA	1000	1000	1000
Isoeugenol	NA	NA	1000	NA	NA	NA
Penicillin/erythromycin	125	250	250	250	250	62.5

*NA* no activity at the concentrations analyzed

Among the compounds tested, **8** and **16** showed the highest activity in inhibiting the strains. Compound **16** had the highest activity of all the derivatives involved in this study, and regarding *K. pneumoniae* and *B. cereus* strains, it was two times more active than eugenol. In contrast, compound **8** exhibited, in comparative terms, strong antibiotic activity against the *E. coli* strain, where the eugenol itself is inactive. Whereas epoxide **16** from eugenol showed strong relative activity, the corresponding acetate **17** showed a marginal effect.

In previous work [35], an MIC of 1200 μg/mL was recorded for eugenol against *S. aureus* bacteria, consistent with an MIC of 1000 μg/mL determined in the present study. These comparative data show that, like eugenol, several of its derivatives have a promising antimicrobial potential.

The antioxidant activity of eugenol derivatives was evaluated with DPPH (2,2-diphenyl-1-picrylhydrazyl). Radical scavenging activity is one of the most widely used methods for screening the antioxidant activity of substances. The ability to capture free radicals by the eugenol derivatives (**1**–**19**) against DPPH was expressed as IC_50_, which represents the concentration required to capture 50% of the radicals in the medium. As positive controls, Trolox and gallic acid were used. Phenolic compounds, such as eugenol, have the facility of transferring electrons or hydrogen atoms by neutralizing free radicals, that is, by blocking the oxidative process [10, 36]. The results (Table 3) showed that all the derivatives (**1**–**19**) presented higher IC_50_ than eugenol, that is, the structural modifications resulted in substances with lower antioxidant effects. All derivatives (**1**–**11, 13, 15, 17, 20** and **21**) produced by the esterification reaction on the hydroxyl group showed a strong reduction in antioxidant activity, as expected [27, 37, 38].

**Table 3:** Inhibitory concentration of 50% (IC_50_) of the free radicals presented by the eugenol derivatives

Substance	IC_50_ (μg/mL)
**1**	> 100
**2**	> 100
**3**	> 100
**4**	> 100
**5**	> 100
**6**	> 100
**7**	> 100
**8**	> 100
**9**	> 100
**10**	> 100
**11**	> 100
**12**	51.12
**13**	> 100
**14**	20
**15**	> 100
**16**	19.3
**17**	> 100
**18**	32
**19**	30.37
Methyl eugenol	> 100
Isoeugenol	50.7
Eugenol	4.38
Gallic acid	0.64
Trolox	16

In the specific case of eugenol, the relationship between the hydroxyl group and the antioxidant action was observed in a previous study [26] through derivatives **2**, **4**, **5**, **6**, and **9**, in which all presented IC_50_ is lower than eugenol.

On the other hand, the chemical modification in the double bond, in the case of the derivatives **12**, **14**, **16**, **18**, and **19**, also caused reduction in the antioxidant capacity against the radical DPPH, however, much lower than that caused by the esterification of the hydroxyl group. Thus, derivatives **16** (IC_50_ 19.3 μg/mL) and **18** (IC_50_ 32 μg/mL), for example, showed antioxidant action close to the Trolox standard (IC_50_ 16 μg/mL).

Derivatives **12**, **14**, **16**, **18**, and **19**, with higher antioxidant action than the others, have a structural characteristic capable of enhancing this action. Although with IC_50_ values higher than eugenol, the results reflect the behavior of the substances in vitro; however, in living biological systems, the antioxidant activity varies, among others, with factors such as the reduction potential in the medium, the displacement capacity of the radical structure formed, the ability to complex transition metals involved in the oxidative process, access to the site of action according to hydrophilicity or lipophilicity, and its partition coefficient [39, 40]. The partition coefficient is closely related to the hydrophilic (or hydrophobic) character of the molecule. In the case of derivatives **12**, **14**, **16**, **18**, and **19**, although less active than eugenol, the hydrophilicity is substantially different, especially for **12** and **14**, which have additional hydroxyl groups allowing a higher degree of hydration and, consequently, greater interaction in aqueous media.

## Conclusions

It was possible to demonstrate that structural modifications in the eugenol molecule resulted in some potentially antibacterial substances (e.g., **8**, **15**, **16**). In addition, various derivatives (**9**, **10**, **12**, **13**, **14**, **15**, **16**, **17**, and **18**) have greater power in inhibiting the growth of certain strains regarding eugenol, as in the case of *Streptococcus* bacteria.

Regarding the antioxidant capacity of the derivatives, the study contributed to make an empirical evaluation of the structure–activity relationship, being observed that the hydroxyl group is decisive in inhibiting the propagation of free radicals. On the other hand, changes in the olefinic bond, although resulting in a slight reduction in the capacity to capture DPPH radicals, and the increase in the hydrophilic character can compensate and contribute as a differential in the antioxidant action.

## Experimental

### General methods

GC–MS analyses were performed using a Shimadzu QP2010SE Plus instrument equipped with a Rtx^®^-5MS (5% phenyl)-dimethylpolysiloxane capillary column (30 m × 0.25 mm) with a film thickness of 0.1 µm using He as carrier gas (1.0 mL/min) in split mode; the injector and detector temperatures were 240 and 280 °C, respectively; column temperature was programmed at 5 °C/min from 60 to 80 °C (3 min), then at 30 °C/min to 280 °C (10 min). Mass spectra were recorded on a Shimadzu QP2010SE apparatus operating in electron impact mode at 70 eV (*scan* mode analysis). ^1^H NMR spectra were recorded on a Bruker DPX 300 (300 MHz) and a Bruker DRX 500 (500 MHz) NMR, using CDCl_3_ solutions and TMS as internal standard.

### Synthesis of eugenol derivatives: (**1**–**3**)

In separate experiments, eugenol (328 mg, 2 mmol) was mixed with acetic anhydride (712 mg, 6 mmol), butanoic anhydride (948 mg, 6 mmol), and hexanoic anhydride (1284 mg, 6 mmol). In each mixture, 2 mL of pyridine was added, followed by stirring of the resulting solutions for 24 h at room temperature. At the end of this period, EtOAc (20 mL) was added to the reaction medium, which was then partitioned with a 20% (w/v) aqueous solution of CuSO_4_·5H_2_O (3 × 30 mL). After separation of the EtOAc and H_2_O phases, the organic was washed with saturated NaCl solution (3 × 10 mL) and dried with anhydrous Na_2_SO_4_. The solvent was evaporated under reduced pressure to obtain the compounds **1** (362 mg, 1.76 mmol, 88% yield), **2** (337 mg, 1.44 mmol, 72% yield), and **3** (314.4 mg, 1.2 mmol, 60% yield). **4**–**11**: In individual experiments, a mixture of eugenol (328 mg, 2 mmol), DMAP (50 mg, 0.4 mmol), and DCC (118 mg, 3 mmol) was added to benzoic acid (366 mg, 3 mmol), 4-methylbenzoic acid (432 mg, 3 mmol), 4-fluorobenzoic acid (420 mg, 3 mmol), 4-chlorobenzoic acid (469.5 mg, 3 mmol), 4-bromobenzoic acid (603 mg, 3 mmol), 4-nitrobenzoic acid (501 mg, 3 mmol), *trans*-cinnamic acid (444 mg, 3 mmol), and 2-(4-isopropylphenyl)propanoic acid (ibuprofen, 618 mg, 3 mmol) in CH_2_Cl_2_ (5 mL). The reaction mixtures were stirred at room temperature for 24 h. At the end of this period, each reaction mixture was filtered and the liquid phases were washed successively with 5% (m/v) HCl (2 × 5 mL), 5% NaHCO_3_ (w/v; 3 × 5 mL), and H_2_O (3 × 5 mL). Finally, after drying with anhydrous Na_2_SO_4_, the organic phases were evaporated under reduced pressure to afford **4** (353 mg, 1.32 mmol, 66% yield), **5** (350 mg, 1.24 mmol, 62% yield), **6** (457.6 mg, 1.6 mmol, 80% yield), **7** (453 mg, 1.5 mmol, 75% yield), **8** (484 mg, 1.4 mmol, 70% yield), **9** (438 mg, 1.4 mmol, 70% yield), **10** (376 mg, 1.28 mmol, 64% yield), and **11** (422 mg, 1.2 mmol, 60% yield) [41]. **12**: To a stirred yellow-colored solution of HgSO_4_ (1483 mg, 5 mmol) in water (5 mL) and THF (5 mL) was added eugenol (820 mg, 5 mmol). After disappearance of the yellow coloration (ca. 4 h) a mixture of 3 M aqueous NaOH (5 mL) and 0.5 M NaBH_4_ (5 mL) was added, followed by vigorous stirring for 30 min. At the end of this period, the reaction mixture was poured into a saturated aqueous solution of NaCl (20 mL) and extracted with THF (3 × 5 mL). The combined extracts were dried in anhydrous Na_2_SO_4_ and concentrated to give a residue (637 mg) which was chromatographed over Si gel column to give **12** (318 mg, 1.75 mmol, 35% yield) [42]. **13**: A solution of **12** (182 mg, 1 mmol) in a mixture of Ac_2_O (612 mg, 6 mmol) and C_5_H_5_N (1 mL) was stirred for 24 h at room temperature. At the end of this period [complete acetylation was indicated by TLC (Si gel, hexane–EtOAc 7:3)], EtOAC (20 mL) was added to the reaction medium, which was then partitioned with a 20% (w/v) aqueous solution of CuSO_4_·5H_2_O (3 × 5 mL). After separation of the EtOAc and H_2_O phases, the organic phase was dried with anhydrous Na_2_SO_4_ and the solvent evaporated under reduced pressure **13** (127 mg, 0.48 mmol, 48% yield) [43]. **14**: Eugenol (820 mg, 5 mmol) in CH_2_Cl_2_ (5 mL) was added dropwise to *m*-chloroperbenzoic acid (1.30 g) in CH_2_Cl_2_ (15 mL) at 25 °C. After stirring for 24 h, 10% aq. Na_2_SO_3_ (10 mL) was added to the mixture and the solution was washed two times with 5% NaHCO_3_ (25 mL). The CH_2_Cl_2_ layer was dried (Na_2_SO_4_) and concentrated [44]. The reaction product (360 mg, 2 mmol) in 20% NaOH (10 mL) was heated at 80 °C for 2 h. The reaction mixture was cooled (28 °C), diluted with water, and neutralized with 10% HCl to pH 7.0. The water was removed under reduced pressure and the resultant mass was extracted with anhydrous EtOH (5 × 10 mL). The ethanolic solution was filtered, dried with anhydrous Na_2_SO_4_, and concentrated under reduced pressure to give a crude product which was subsequently chromatographed over Si gel column **14** (435.6 mg, 2.2 mmol, 44% yield). **15**: Product **14** (198 mg, 1 mmol) was treated with Ac_2_O (1020 mg, 10 mmol) and anhydrous pyridine (3 mL). The resultant solution was stirred at room temperature for 24 h. After this time, the reaction was complete, as indicated by TLC [Si gel, hexane–EtOAc (7:3)], and EtOAC (20 mL) was added to the reaction medium, which was then partitioned with a 20% (w/v) aqueous solution of CuSO_4_·5H_2_O (5 × 10 mL). After separation of the EtOAc and H_2_O phases, the organic was dried with anhydrous Na_2_SO_4_ and the solvent evaporated under reduced pressure to afford **15** (110 mg, 0.34 mmol, 34% yield). **16**: Eugenol (820 mg) in CH_2_Cl_2_ (5 mL) was added dropwise to *m*-chloroperbenzoic acid (1.30 g) in CH_2_Cl_2_ (15 mL) at 25 °C. After stirring for 24 h, 10% aq. Na_2_SO_3_ (10 mL) was added to the mixture and the solution was washed two times with 5% NaHCO_3_ (25 mL). The CH_2_Cl_2_ layer was dried with Na_2_SO_4_ and concentrated. The residue was purified by silica gel CC with hexane/ethyl acetate (90:10) to give **7** (1.00 g) and **16** (328 mg, 2 mmol, 40% yield). **17:** A solution of **16** (180 mg, 1 mmol) in a mixture of Ac_2_O (306 mg, 3 mmol) and C_5_H_5_N (0.5 mL) was stirred under ice bath for 3 h. After this time, the reaction was complete, as indicated by TLC [Si gel, hexane–EtOAc (8:2)], and EtOAc (20 mL) was added to the reaction medium, which was then partitioned with a 20% (w/v) aqueous solution of CuSO_4_·5H_2_O (3 × 5 mL). After separation of the EtOAc and H_2_O phases, the organic was dried with anhydrous Na_2_SO_4_ and the solvent evaporated under reduced pressure to afford **17** (138 mg, 0.62 mmol, 62% yield). **18**: To a stirred solution of ZnCl_2_ (0.634 g, 4.95 mmol) in acetone (5 mL) at 0 °C was added over a period of 10 min the compound **14** (396 mg, 2 mmol). The reaction mixture was then warmed at 30 °C, where stirring was continued for an additional time of 24 h. The reaction was quenched by the addition of a mixture of CHCl_3_ (10 mL) and saturated aqueous NaCl (10 mL) and extracted with CHCl_3_ (3 × 10 mL). The organic phases were dried with anhydrous Na_2_SO_4_ and the solvent evaporated under reduced pressure to afford **18** (285.6 mg, 1.2 mmol, 60% yield) [45]. **19**: Compound **18** (238 mg, 1 mmol) was treated with Ac_2_O (306 mg, 3 mmol) and anhydrous pyridine (1 mL). The resultant solution was stirred at room temperature for 18 h. After this time, the reaction was complete, as indicated by TLC [Si gel, hexane–EtOAc (8:2)], and EtOAC (20 mL) was added to the reaction medium, which was then partitioned with a 20% (w/v) aqueous solution of CuSO_4_·5H_2_O (3 × 10 mL). After separation of the EtOAc and H_2_O phases, the organic was dried with anhydrous Na_2_SO_4_ and the solvent evaporated under reduced pressure to afford **19** (196 mg, 0.7 mmol, 62% yield). The characterization of derivatives is detailed in Additional file 1.

### Antibacterial activity of eugenol derivatives by inhibition zone (disk diffusion)

Quantitative and qualitative antibacterial screening was performed in the Federal Institute of Education, Science and Technology of Rio Grande do Norte, Apodi campus. The procedure consisted of testing the pure compounds against the following microorganisms, obtained from according to norms approved by the National Sanitary Surveillance Agency^32^: *Pseudomonas aeruginosa, Escherichia coli, Staphylococcus aureus, Streptococcus, Klebsiella pneumoniae, Bacillus cereus*. The bacterial strains were replicated in Muller Hilton agar medium (MH) and incubated for 24 h at 35 °C. Plates for the assay were prepared by dispersing the Muller Hilton agar medium in sterile Petri dishes and the bacteria were incubated at 35 °C. Then, with the help of a flame-sterilized platinum handle, the bacterial cells were transferred to a sterile test tube containing 0.85% NaCl solution until reaching an absorption between 0.08 and 0.10 in a spectrophotometer at the wavelength of 625 nm (corresponding to approximately 1 × 10^8^ cells). In the process, a sterile swab was soaked in the bacterial suspension and compressed into the whole assay to avoid excess material. This was then applied in uniform motions on the culture medium until the entire surface was filled. For the disks, 20 µL of the pure compounds was added at concentrations of 10 mg/mL in DMSO/water (1:1). The plates prepared as described were incubated at 35 °C. The antimicrobial activity was recorded as the width (in mm) of the inhibition zone after 24 h of incubation. A standard antibacterial agent (amikacin—30 mcg) was included in each assay as positive control.

### Antibacterial activity of eugenol derivatives: minimum inhibitory concentration (MIC)

The antibacterial activity of eugenol derivatives was determined by the microdilution method, recommended by the National Committee for Clinical and Laboratory Standard M7-A6^32^. The procedure consisted of testing the pure compounds in six standard Gram (+) and Gram (−) bacteriological strains: *P. aeruginosa*, *E. coli*, *S. aureus*, *Streptococcus*, *K. pneumoniae*, *B. cereus*). The Muller Hilton Broth (MHB) was used as medium for the bacterial growth (35 °C, 24 h). After this time, the culture of each bacterial species in the MHB was diluted in the same medium to a concentration of approximately 1 × 10^8^ CFU/mL (0.5 NTU—McFarland scale). Each suspension was further diluted to a final concentration of 1 × 10^6^ NTU in NaCl solution (0.85%) with 10% MHB. A volume of 100 μL of each suspension was distributed into the wells of the microplates resulting in a final inoculum concentration of 5 × 10^5^ NTU. The initial solution of the eugenol derivatives was made using 10 mg of each dissolved in 1 mL of DMSO/water (1:1). From this concentration (10 mg/mL), several dilutions were made in distilled water in order to obtain a stock solution of 2000 µg/mL. Further serial dilutions were performed in microplates by addition of MHB (100 µL) to reach a final concentration in the range of 7.8–1000 μg/mL. All the experiments were performed in triplicate and the microdilution trays were incubated in bacteriological oven at 35 °C for 24 h. After this period, the antibacterial activity was detected using a colorimetric method by adding 25 µL of the resazurin staining (0.01%) aqueous solution in each well of the microplate. The minimum inhibitory concentration (MIC) was defined as the lowest extract concentration that can inhibit bacterial growth, as indicated by resazurin staining (dead bacterial cells are not able to change the staining color by visual observation—blue to red).

### Free radical scavenging activity (DPPH Assay)

The free radical scavenging activity was determined by the DPPH assay [46, 47]. 2 mL of various concentrations (10, 20, 30, 50, 70, 100 µg/mL) of the compounds in methanol was added to 2 mL of a methanol solution of 6.6 × 10^−2^ mM DPPH. The decrease in absorbance was determined at 517 nm at room temperature at 0 min, 1 min, and every 5 min for 1 h. For each antioxidant concentration tested, the reaction kinetics was plotted and from these graphs, the absorbance was read after 30 min. Inhibition of the DPPH radical in percent was calculated according to Eq. 1:

Equation 1: Inhibition of the DPPH radical.

1 $${\text{I (\%)}} = 100\cdot\left(1- {\frac{{\left( { A{\text{sample }} - A{\text{blank}}} \right)}}{{A{\text{blank}}}}} \right)$$

where *A*blank is the absorbance of the control and *A*sample is the absorbance of the sample. Sample concentration providing 50% inhibition (IC_50_) was calculated from the graph plotting inhibition percentage against sample concentration. Tests were carried out in triplicate, and Trolox and gallic acid were used as positive controls.

## Additional file

**Additional file 1.** Additional material.
