# Supplementary material for: Eugenol derivatives: synthesis, characterization, and evaluation of antibacterial and antioxidant activities
Source: Chem Cent J. 2018 Apr 3;12:34. doi: 10.1186/s13065-018-0407-4 (PMC5880794; doi:10.1186/s13065-018-0407-4)
Supplement: Supplementary file 1 — Additional file 1. Additional material. [file 13065_2018_407_MOESM1_ESM.docx]

**Additional material**

**Eugenol derivatives: obtention, characterization and evaluation of antibacterial and antioxidant activities**

**Charactrization of derivatives (1-19)**

**1**: **CG-MS**: coluna capilar Rtx®-5MS, T_R_  12.10 min. MS m/z 206 (M^+^), 164 (M-42, 100), 149, 131, 91, 43. **^1^H NMR** (300 MHz, CDCl_3_): δ 2.31 (s, 3H), 3.40 (d, 6.6 Hz, 2H), 3.83 (s, 3H, OCH_3_), 5.10 (sl, 1H), 5.50 (dl, 1H), 6.00 (m, 1H), 6.78 (dl, 8.0 Hz, 1H), 6.79 (sl, 1H), 6.97 (d, 7.1 Hz, 1H). **^13^C NMR** (75 MHz, CDCl_3_): δ 20.82; 40.26; 55.99; 112.94; 116.31; 120.85; 122.70; 137.22; 138.22; 139.18; 151.07; 169.37.

**2**: **CG-MS**:coluna capilar Rtx®-5MS, T_R_  12.50 min. MS m/z 234 (M^+^), 164 (M-70, 100), 149, 131, 91, 43. : **^1^H NMR** (300 MHz, CDCl_3_): δ 1.07 (t, 7.4 Hz, 3H), 1.80 (m, 2H), 2.58 (t, 7.3 Hz, 2H), 3.39 (d, 6.7 Hz, 2H), 3.82 (s, 3H, OCH_3_), 5.10 (sl, 1H), 5.51 (dl, 1H), 6.00 (m, 1H), 6.77 (dl, 1H), 6.79 (sl, 1H), 6.97 (d, 7.9 Hz, 1H). **^13^C NMR** (75 MHz, CDCl_3_): δ 13.75; 18.77; 36.10; 40.26; 55.98; 112.97; 116.26; 120.85; 122.75; 137.28; 138.33; 139.02; 151.13; 172.00.

**3**: **CG-MS**: coluna capilar Rtx®-5MS, T_R_  13.30 min. MS m/z 262 (M^+^), 164 (M-98, 100), 149, 132, 121, 104, 91, 43. **^1^H NMR** (300 MHz, CDCl_3_): δ 0.94 (t, 6.9 Hz, 3H), 1.41 (m, 4H), 1.76 (m, 2H), 2.57 (t, 7.4 Hz, 2H), 3.38 (d, 6.7 Hz, 2H), 3.82 (s, 3H, OCH_3_), 5.08 (sl, 1H), 5.14 (dl, 1H), , 6.00 (m, 1H), 6.77 (dl, 1H), 6.79 (sl, 1H), 6.94 (d, 7.9 Hz, 1H). **^13^C NMR** (75 MHz, CDCl_3_): δ 14.13; 22.52; 24.94; 31.43; 34.22; 40.28; 55.98; 112.97; 116.27; 120.86; 122.74; 137.29; 138.34; 139.02; 151.14; 172.22.

**4**: **CG-MS**: coluna capilar Rtx®-5MS, T_R_ 14.70 min. MS m/z 268 (M^+^), 105 (M-163, 100), 77, 51. **^1^H NMR** (300 MHz, CDCl_3_): δ 3.42 (d, 6.6 Hz, 2H), 3.81 (s, 3H, OCH_3_), 5.11 (sl, 1H), 5.16 (dl, 1H), 6.00 (m, 1H), 6.83 (dl, 9.0 Hz, 1H), 6.85 (sl, 1H), 7.08 (d, 7.8 Hz, 1H), 7.51 (m, 2H), 7.61 (tl, 1H), 8.06 (d, 8.1 Hz, 2H). **^13^C NMR** (75 MHz, CDCl_3_): δ 40.30; 56.09; 113.14; 116.30; 120.94; 122.85; 128.48; 130.47; 132.96; 133.54; 137.30; 138.47; 139.23; 151.34; 165.05.

**5**: **CG-MS**: coluna capilar Rtx®-5MS, T_R_ 15.28 min. MS m/z 282 (M^+^), 119 (M-163, 100), 91, 65. **^1^H NMR** (300 MHz, CDCl_3_): δ 2.42 (s, 3H), 3.42 (d, 6.6 Hz, 2H), 3.81 (s, 3H, OCH_3_), 5.10 (sl, 1H), 5.15 (dl, 1H), 6.00 (m, 1H), 6.82 (dl, 1H), 6.84 (sl, 1H), 7.07 (d, 7.8 Hz, 1H), 7.29 (d, 7.9 Hz, 2H), 8.11 (d, 7.9 Hz, 2H). **^13^C NMR** (75 MHz, CDCl_3_): δ 21.91; 40.31; 56.11; 113.15; 116.28; 120.94; 122.92; 127.04, 129.38; 130.53; 137.34; 138.57; 139.12; 144.32; 151.41; 165.12.

**6**: **CG-MS**: coluna capilar Rtx®-5MS, T_R_ 14.25 min. MS m/z 286 (M^+^), 123/124 (M-163, 100), 103, 95, 51. **^1^H NMR** (300 MHz, CDCl_3_): δ 3.42 (d, 6.2 Hz, 2H), 3.82 (s, 3H, OCH_3_), 5.10 (sl, 1H), 5.14 (dl, 1H), 6.00 (m, 1H), 6.82 (dl,1H), 6.85 (sl, 1H), 7.07 (d, 7.8 Hz, 1H), 7.19 (tl, 2H), 8.15 (dtl, 2H). **^13^C NMR** (75 MHz, CDCl_3_): δ 40.30; 56.09; 113.13; 115.71; 116.00; 120.96; 122.81; 126.05, 133.15; 137.26; 138.35; 139.37; 151.29; 164.13; 164.60.

**7**: **CG-MS**: coluna capilar Rtx®-5MS, T_R_ 15.20 min. MS m/z 302 (M^+^), 139/141 (M-163, 100), 111, 91, 51. **^1^H NMR** (300 MHz, CDCl_3_): δ 3.42 (d, 6.6 Hz, 2H), 3.81 (s, 3H, OCH_3_), 5.07 (sl, 1H), 5.13 (dl, 1H), 6.00 (m, 1H), 6.82 (dl, 9.0 Hz, 1H), 6.85 (sl, 1H), 7.06 (d, 8.1 Hz, 1H), 7.50 (d, 8.2 Hz, 2H), 8.15 (d, 8.2 Hz, 2H). **^13^C NMR** (75 MHz, CDCl_3_): δ 40.30; 56.06; 113.08; 116.37; 120.94; 122.74; 128.21, 129.03; 131.85; 137.23; 138.25; 139.41; 140.09; 151.21; 164.22.

**8**: **CG-MS**: coluna capilar Rtx®-5MS, T_R_ 15.20 min. MS m/z 302 (M^+^), 139/141 (M-163, 100), 111, 91, 51. **^1^H NMR** (300 MHz, CDCl_3_): δ 3.42 (d, 6.7 Hz, 2H), 3.81 (s, 3H, OCH_3_), 5.10 (sl, 1H), 5.14 (dl, 7.83, 1H), 6.00 (m, 1H), 6.82 (dl, 9.2 Hz, 1H), 6.84 (sl, 1H), 7.06 (d, 7.9 Hz, 1H), 7.65 (d, 8.6 Hz, 2H), 8.08 (d, 8.6 Hz, 2H). **^13^C NMR** (75 MHz, CDCl_3_): δ 40.29; 56.07; 113.11; 116.37; 120.95; 122.73; 128.68, 128.77; 131.97; 137.22; 138.26; 139.43; 151.21; 164.36.

**9**: **CG-MS**: coluna capilar Rtx®-5MS, T_R_  16.65 min. MS m/z 313 (M^+^), 150 (M-163, 100), 134, 120, 104, 92, 76, 65, 50, 41. **^1^H NMR** (300 MHz, CDCl_3_): δ 3.42 (d, 6.6 Hz, 2H), 3.82 (s, 3H, OCH_3_), 5.07 (sl, 1H), 5.12 (dl, 1H), 6.00 (m, 1H), 6.71 (dl, 1H), 6.84 (sl, 1H), 7.08 (d, 7.9 Hz, 1H), 8.35 (d, 8.9 Hz, 2H), 8.38 (d, 8.9 Hz, 2H). **^13^C NMR** (75 MHz, CDCl_3_): δ 40.30; 56.07 113.14; 116.49; 121.01; 122.54; 123.82; 130.85; 131.58; 137.12; 138.02; 139.85; 148.51; 151.03; 163.21.

**10**: **CG-MS**: coluna capilar Rtx®-5MS, T_R_  16.40 min. MS m/z 294 (M^+^), 131 (M-163, 100), 103, 77, 65, 51. **^1^H NMR** (300 MHz, CDCl_3_): δ 3.42 (d, 6.6 Hz, 2H), 3.85 (s, 3H, OCH_3_), 5.12 (sl, 1H), 5.16 (dl, 1H), 6.00 (m, 1H), 6.82 (dl, 1H), 6.84 (sl, 1H), 7.05 (d, 7.8 Hz, 1H), 7.06 (d, 15.9 Hz, 1H), 7.43 (m, 2H), 7.43 (m, 1H), 7.59 (tl, 2H), 7.90 (d, 15.9 Hz, 1H). **^13^C NMR** (75 MHz, CDCl_3_): δ 40.28; 56.04; 112.99; 116.31; 117.31; 120.89; 122.82; 128.45, 129.11; 130.73; 134.49; 137.26; 138.22; 139.15; 146.58; 151.22; 165.26.

**11**: **CG-MS**: coluna capilar Rtx®-5MS, T_R_  16.60 min. MS m/z 352 (M^+^), 161/188 (M-163, 100), 145, 117, 91, 57, 43. **^1^H NMR** (300 MHz, CDCl_3_): δ 0.95 (d, 6.5 Hz, 6H), 1.65 (d, 7.1 Hz, 3H), 1.91 (m, 1H), 2.50 (d, 7.1 Hz, 2H), 3.38 (d, 6.6 Hz, 2H), 3.73 (s, 3H, OCH_3_), 4.00 (q, 7.1 Hz, 1H), 5.08 (sl, 1H), 5.13 (dl, 1H), 6.00 (m, 1H), 6.72 (sl, 1H) 6.74 (dl, 1H), 6.88 (d, 7.8 Hz, 1H), 7.17 (d, 7.9 Hz, 2H), 7.32 (d, 7.9 Hz, 2H). **^13^C NMR** (75 MHz, CDCl_3_): δ 18.94; 22.58; 30.39; 40.23; 45.22; 45.28; 55.94; 113.12; 116.22; 120.82; 122.55; 127.61, 129.46; 137.31; 137.71; 138.52; 138.99; 140.74; 151.21; 173.12.

**12**: **CG-MS**: coluna capilar Rtx®-5MS, T_R_  12.00 min. MS m/z 182 (M^+^), 137 (M-45, 100), 122, 94, 45. **^1^H NMR** (300 MHz, CDCl_3_): δ 1.24 (d, 6.2 Hz, 3H),1.74 (sl, 1H, OH); 2.60 (dd, 13.6 e 5.2 Hz, 1H), 2.73 (dd, 13.6 e 4.6 Hz, 1H), 3.88 (s, 3H, OCH_3_), 5.68 (sl, 1H, OH); 6.69 (dl, 1H), 6.72 (sl, 1H), 6.85 (d, 7.6 Hz, 1H). **^13^C NMR** (75 MHz, CDCl_3_): δ 22.85; 45.60; 56.07; 69.14; 112.11; 114.68; 122.18; 130.48; 144.50; 146.76.

**13**: **CG-MS**: coluna capilar Rtx®-5MS, T_R_  13.10 min. MS m/z 266 (M^+^), 164 (M-102, 100), 224, 206, 137, 122, 77, 65, 43. **^1^H NMR** (300 MHz, CDCl_3_): δ 1.23 (d, 6.3 Hz, 3H), 2.01 (s, 3H), 2.30 (s, 3H), 2.71 (dd, 13.7 e 6.5 Hz, 1H), 2.91 (dd, 13.7 e 6.6 Hz, 1H), 3.82 (s, 3H, OCH_3_), 5.11 (m, 1H); 6.76 (d, 8.0 Hz, 1H), 6.80 (sl, 1H), 6.94 (d, 8.0 Hz, 1H). **^13^C NMR** (75 MHz, CDCl_3_): δ 19.65; 20.80; 21.45; 42.28; 56.05; 71.52; 113.70; 121.77; 122.69; 136.73; 138.61; 151.04; 169.29; 170.78.

**14**: **CG-MS**: coluna capilar Rtx®-5MS, T_R_  13.11 min. MS m/z 198 (M^+^), 137 (M-61, 100), 107, 77, 65, 39. **^1^H NMR** (300 MHz, CDCl_3_): δ 2.69 (dd, 13.7 e 4.1 Hz, 1H), 2.73 (dd, 13.7 e 6.4 Hz, 1H), 3.42 (dd, 11.1 e 6.2 Hz, 1H), 3.46 (dd, 11.1 e 4.1 Hz, 1H), 3.83 (s, 3H, OCH_3_), 3.85 (m, 1H); 6.65 (dd, 8.0 e 1.8 Hz, 1H), 6.71 (d, 8.8 Hz, 1H), 6.81 (d, 1.7 Hz, 1H). **^13^C NMR** (75 MHz, CDCl_3_): δ 40.63; 56.50; 74.79; 114.29; 116.17; 123.02; 131.67; 145.99; 148.88.

**15**: **CG-MS**: coluna capilar Rtx®-5MS, T_R_ 14.27 min. MS m/z 324 (M^+^), 222 (M-102, 100), 179, 163, 137, 124, 91, 65, 43. **^1^H NMR** (300 MHz, CDCl_3_): δ 2.03 (s, 3H), 2.07 (s, 3H), 2.29 (s, 3H), 2.88 (m, 2H), 3.80 (s, 3H, OCH_3_), 4.03 (dd, 12.0 e 6.1 Hz, 1H), 4.24 (dd, 12.0 e 3.2 Hz, 1H), 5.25 (m, 1H); 6.77 (d, 8.0 Hz, 1H), 6.81 (sl, 1H), 6.93 (d, 8.0 Hz, 1H). **^13^C NMR** (75 MHz, CDCl_3_): δ 20.78; 20.89; 21.16; 37.10; 56.02; 64.34; 72.05; 113.49; 121.64; 122.91; 135.40; 138.80; 151.17; 169.17; 170.47; 170.81.

**16**: **CG-MS**: coluna capilar Rtx®-5MS, T_R_ 12.40 min. MS m/z 180 (M^+^), 137 (M-43, 100), 165, 151, 122, 107, 91, 77, 65, 51. **^1^H NMR** (300 MHz, CDCl_3_): δ 2.80 (dl, 5.0 Hz, 2H), 2.81 (dl, 5.0 Hz, 2H), 3.13 (m, 1H), 3.89 (s, 3H, OCH_3_), 5.66 (sl, 1H, OH); 6.74 (dl, 8.0 Hz, 1H), 6.76 (sl, 1H), 6.86 (d, 7.9 Hz, 1H). **^13^C NMR** (75 MHz, CDCl_3_): δ 38.54; 46.96; 52.87; 56.10; 111.84; 114.59; 121.84; 129.21; 144.64; 146.73.

**17**: **CG-MS**: coluna capilar Rtx®-5MS, T_R_ 13.30 min. MS m/z 222 (M^+^), 180 (M-42, 100), 162, 124, 91, 77, 43. **^1^H NMR** (300 MHz, CDCl_3_): δ 0,94 (t, 3H) 1.41 (m, 2H), 1.76 (m, 2H), 2.57 (t, 2H), 3.38 (d, 6.6 Hz, 2H), 3.82 (s, 3H), 5.08 (dd, 1H), 5.14 (dd, 1H), 5.90-6.04 (m, 1H), 6.77 (dd, 1H), 6.79 (d, 1H), 6.94 (d, 8.0Hz, 1H). **^13^C NMR** (75 MHz, CDCl_3_): δ 14.10; 22.50; 24.94; 31.42; 34.22; 40.27; 55.98; 112.96; 116.27; 120.86; 122.73; 137.29; 138.34; 139.02; 151.13; 172.22.

**18**: **CG-MS**: coluna capilar Rtx®-5MS, T_R_ 13.20 min. MS m/z 238 (M^+^), 101 (M-137, 100), 223, 163, 147, 137, 122, 94, 73, 59, 43. **^1^H NMR** (300 MHz, CDCl_3_): δ 1.36 (s, 3H), 1.44 (s, 3H), 2.71 (dd, 13.7 e 7.2 Hz, 1H), 2.93 (dd, 13.7 e 5.9 Hz, 1H), 3.65 (m, 1H), 3.95 (tl, 1H), 3.87 (s, 3H, OCH_3_), 4.28 (m, 1H); 6.68 (dl, 8.1 Hz, 1H), 6.72 (sl, 1H), 6.83 (d, 7.9 Hz, 1H). **^13^C NMR** (75 MHz, CDCl_3_): δ 25.88; 27.18; 39.83; 56.05; 69.07; 77.02; 109.30; 111.99; 114.59; 121.99; 129.52; 144.49; 146.64.

**19**: **CG-MS**: coluna capilar Rtx®-5MS, T_R_ 13.20 min. MS m/z 280 (M^+^), 101 (M-179, 100), 265, 163, 137, 122, 91, 73, 59, 43. **^1^H NMR** (300 MHz, CDCl_3_): δ 0,94 (t, 3H) 1.41 (m, 2H), 1.76 (m, 2H), 2.57 (t, 2H), 3.38 (d, 6.6 Hz, 2H), 3.82 (s, 3H), 5.08 (dd, 1H), 5.14 (dd, 1H), 5.90-6.04 (m, 1H), 6.77 (dd, 1H), 6.79 (d, 1H), 6.94 (d, 8.0Hz, 1H). **^13^C NMR** (75 MHz, CDCl_3_): δ 14.10; 22.50; 24.94; 31.42; 34.22; 40.27; 55.98; 112.96; 116.27; 120.86; 122.73; 137.29; 138.34; 139.02; 151.13; 172.22.
